# QuickStats

**Published:** 2015-03-13

**Authors:** FROM THE NATIONAL CENTER FOR HEALTH STATISTICS

**Figure:**
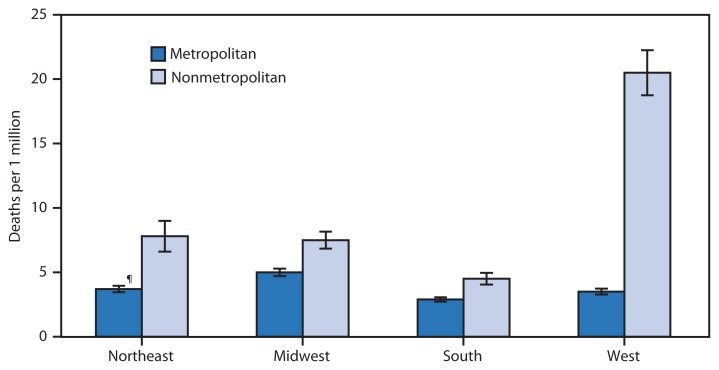
Age-Adjusted Rates for Cold-Related Deaths,* by U.S. Census Region^†^ and Metropolitan Status of Place of Occurrence^§^ — United States, 2010–2013 * Age-adjusted rates per 1 million population; based on the 2000 U.S. standard population. Deaths attributed to exposure to excessive natural cold (X31) (underlying or contributing cause of death), hypothermia (T68) (contributing cause of death), or effect of reduced temperature, unspecified (T69.9) (contributing cause of death), or a combination of these, according to the *International Classification of Diseases, 10th Revision*. Rates computed by place of occurrence. During 2010–2013, 5,809 cold-related deaths occurred in the United States. ^†^ *Northeast:* Connecticut, Maine, Massachusetts, New Hampshire, Rhode Island, New Jersey, New York, Pennsylvania, and Vermont; *Midwest:* Illinois, Indiana, Iowa, Kansas, Michigan, Minnesota, Missouri, Nebraska, North Dakota, Ohio, South Dakota, and Wisconsin; *South:* Alabama, Arkansas, Delaware, Florida, Georgia, Kentucky, Louisiana, Mississippi, Maryland, North Carolina, Oklahoma, South Carolina, Virginia, Tennessee, Texas, West Virginia, and District of Columbia; *West:* Alaska, Arizona, California, Colorado, Hawaii, Idaho, Montana, Nevada, New Mexico, Oregon, Utah, Washington, and Wyoming. ^§^ Counties were classified as metropolitan or nonmetropolitan based on the February 2013 Office of Management and Budget delineation. ^¶^ 95% confidence interval.

In all regions of the United States, cold-related mortality during 2010–2013 was higher in nonmetropolitan areas than in metropolitan areas. Age-adjusted cold-related death rates in nonmetropolitan areas of the West were markedly higher than those in the other regions (20.5 deaths per 1 million population compared with 4.5–7.8). Age-adjusted cold-related death rates in metropolitan areas ranged from 2.9 to 5.0 deaths per 1 million population, with the South having the lowest rate.

**Sources:** National Vital Statistics System. County-level mortality file. Available at http://www.cdc.gov/nchs/deaths.htm and http://wonder.cdc.gov/mcd.html.

**Reported by:** Deborah D. Ingram, PhD, ddingram@cdc.gov, 301-458-4733.

